# Odontogenic orbital cellulitis associated with cavernous sinus thrombosis and pulmonary embolism: a case report

**DOI:** 10.1186/s13256-017-1309-0

**Published:** 2017-06-20

**Authors:** D. Allegrini, S. Reposi, E. Nocerino, A. Pece

**Affiliations:** 1 Eye Unit, Humanitas Gavazzeni Hospital, Humanitas University, Bergamo, Italy; 2 Eye Clinic, Melegnano Hospital, Vizzolo Predabissi, Milan, Italy; 3 Radiology Department, San Paolo Hospital, University of Milan, Milan, Italy

**Keywords:** Odontogenic orbital cellulitis, Cavernous sinus thrombosis, Pulmonary embolism

## Abstract

**Background:**

This case illustrates the importance of prompt assessment and treatment of orbital cellulitis. In fact the ocular signs and symptoms may be associated with systemic complications which should be investigated and identified as soon as possible to avoid a poor prognosis.

**Case presentation:**

A 46-year-old white woman presented to our emergency room with proptosis, ophthalmoplegia, and conjunctival chemosis of her left eye. An ophthalmologist, having diagnosed orbital cellulitis in her left eye, suspected a cavernous sinus thrombosis. Hematochemical and radiological examinations confirmed the cavernous sinus thrombosis and also showed septic pulmonary embolism. A blood culture indicated *Streptococcus constellatus*, which is a member of the *Peptostreptococcus* family, a saprophyte of the oral mucosa that can be pathogenic in immunocompromised persons. The odontogenic origin was then confirmed by dental radiography which showed a maxillary abscess. Her eye signs regressed after antibiotic and anticoagulant therapy.

**Conclusions:**

This complex case shows the importance of a multidisciplinary approach for the management of orbital cellulitis, for the prompt diagnosis and treatment of eye injuries and possible complications, so as to avoid serious and permanent sequelae.

## Background

This case illustrates the importance of prompt assessment and treatment of orbital cellulitis (OC). This infection can lead to abscess formation, blindness, or death and typically originates from sinus infections or cutaneous lesions [1, 2]. However, this patient’s infection originated from a very atypical location: a dental infection. Furthermore, the associated cavernous sinus thrombosis (CST) and pulmonary embolism (PE) are unusual complications. CST and proptosis were caused by retrograde thrombophlebitic diffusion, due to the venous drainage of the head and neck. The lung lesions were caused by septic emboli [3].

This case highlights the need for a multidisciplinary approach and radiological support in the diagnosis and treatment of OC, which can sometimes be the first sign of septic spread.

## Case presentation

A 46-year-old white woman presented to our emergency department with facial pain, eyelid swelling, and proptosis with ophthalmoplegia in her left eye (LE), suggesting unilateral OC, and normal vital signs parameters. These signs had been present for 6 hours and started with ophthalmoplegia by eye movements. She had a history of dyslipidemia and hypertension and was receiving drug therapy; she had no fever, sinusitis, or facial skin infections in the previous days.

**Fig. 1:**
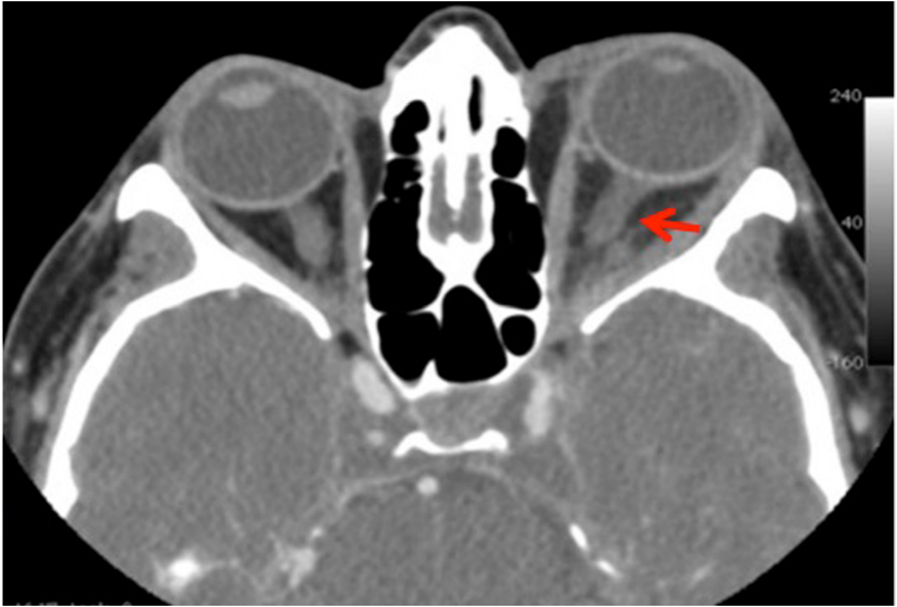
Contrast-enhanced computed tomography. Distended superior ophthalmic vein on the *left side* (*arrow*), with periorbital swelling

**Fig. 2:**
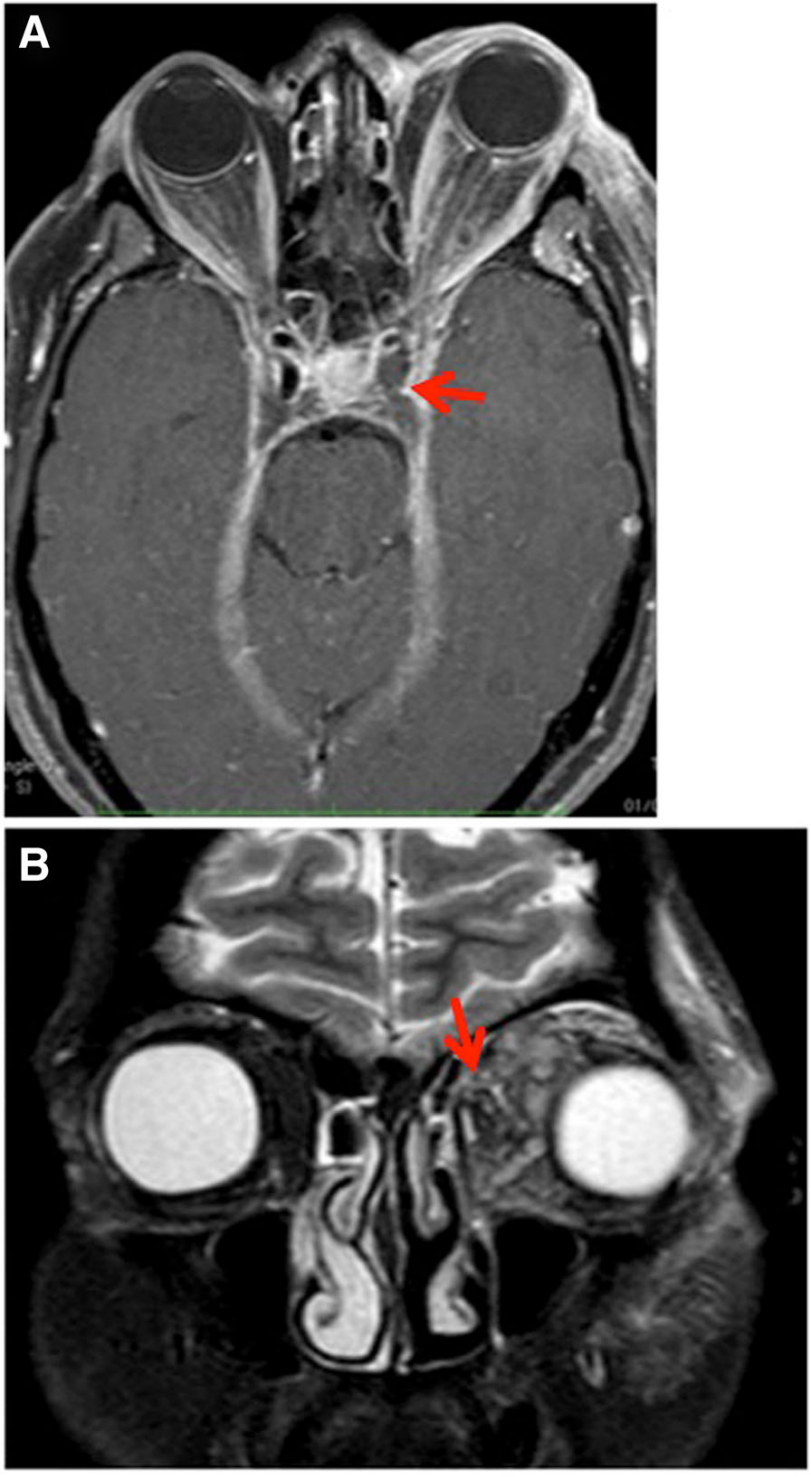
Contrast-enhanced magnetic resonance imaging. **a** A non-fat density filling defect in the *left side* of the cavernous sinus. **b** Coronal plane, periorbital swelling (*arrow*)

Given the absence of other associated issues an ophthalmologic evaluation was initially requested. Her best corrected visual acuity in both eyes was 20/20 with intraocular pressure 15 mmHg, and there was no detectable afferent pupillary defect. Her right eye was normal. Her LE had substantial chemosis and hyperemia of the conjunctiva, with exacerbation of the ophthalmoplegia by eye movements. A fundus examination of her LE was normal. Palpation of her left orbital area generated significant crackles but also revealed major pulsation in the scanned region. An urgent non-contrast-enhanced computed axial tomography (NCCAT) of her brain and orbits showed a dense and distended superior ophthalmic vein on the left side, with periorbital swelling. Contrast-enhanced computed tomography (CECT) confirmed the presence of a thrombus in her superior ophthalmic vein, as a filling defect in the late phase, and also showed a non-fat density filling defect in the ipsilateral part of the cavernous sinus (Fig. 1). Finally, contrast-enhanced magnetic resonance imaging (CEMRI) confirmed the distended cavernous sinus and superior ophthalmic vein with the non-fat density filling defect (Fig. 2).

**Fig. 3:**
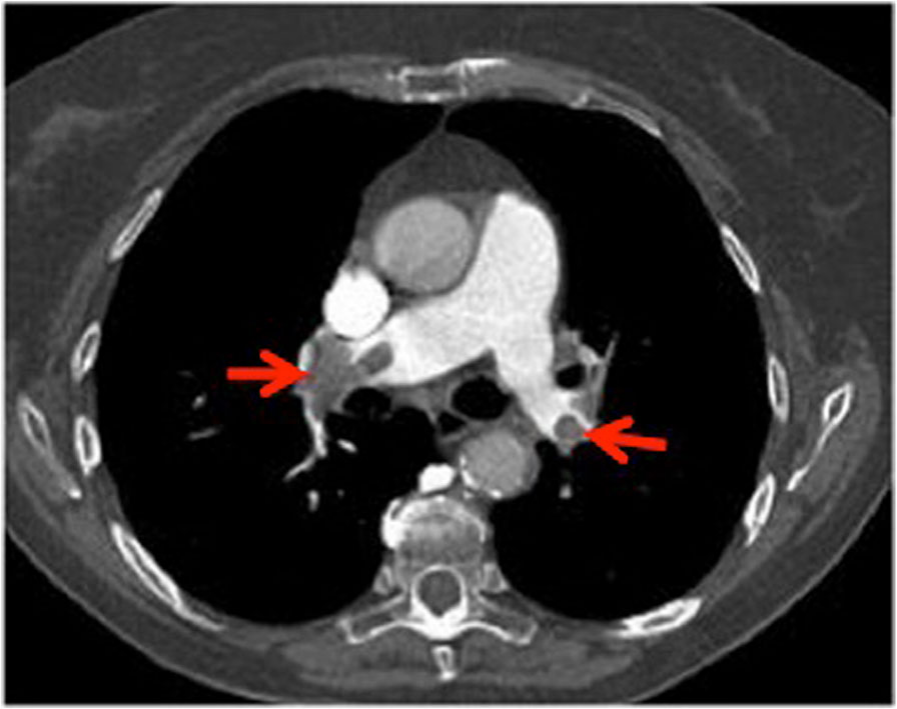
Contrast-enhanced computed tomography pulmonary embolism protocol. Massive bilateral thrombosis in the main pulmonary arteries (*arrows*)

The day after she started to develop dyspnea an arterial blood gas test was done. The findings of hypocapnia and hypoxemia suggested PE, so CECT with the PE protocol was done, showing massive bilateral pulmonary artery thrombosis and a lung infarct in the superior segment of the basal lobe of her right lung (Fig. 3).

She had neutrophilia, with a total white cell count of 18.1 × 10^9^/liter and elevated C-reactive protein of 341 mg/liter. Blood culture was positive for *Streptococcus constellatus*, a member of the *Peptostreptococcus* family, a saprophyte of the oral mucosa that can be pathogenic in immunocompromised persons, but human immunodeficiency virus (HIV) testing was negative. Dental panoramic images confirmed a maxillary abscess.

**Fig. 4:**
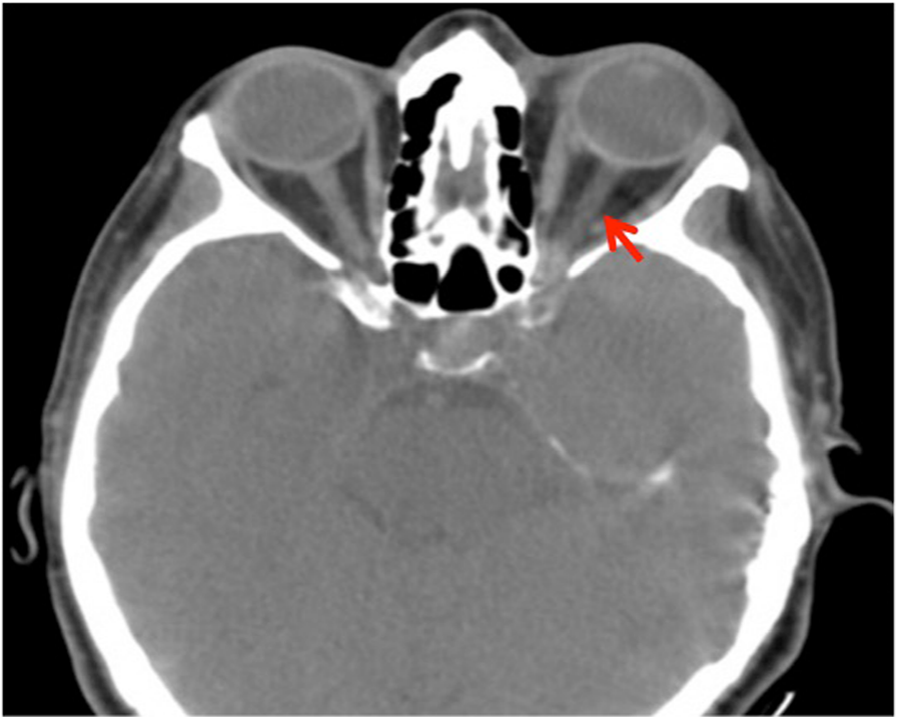
Computed tomography of the brain. Regular diameter of the superior ophthalmic vein on the *left* (arrow) and *regular* signal of the fatty periorbital tissue

She was prescribed a cycle of intravenously administered antibiotics (piperacillin/tazobactam) and anticoagulation therapy with heparin for 10 days, after which she no longer had ophthalmoplegia and periorbital edema. A radiological orbit CECT showed that the diameter of her superior ophthalmic vein on the left and the signal of the fatty periorbital tissue were both normal (Fig. 4). Her white blood cell count normalized. She was discharged from hospital after 15 days with orally administered antibiotic therapy to be taken for 1 month. The affected teeth were extracted after 3 weeks from the onset of ocular symptoms.

At 1-month follow-up she no longer had shortness of breath. A chest CECT check with the PE protocol confirmed there was no evidence of thrombosis in her main pulmonary arteries.

## Discussion

OC can be caused by primary infection of the sinuses, skin, or teeth. Nearly two thirds of cases, 64%, are from primary sinus infection, which is the most common cause of orbital inflammation, and most of these are of bacterial origin [4]; 16% of cases come from cutaneous lesions, such as eczema, furuncles, or facial cellulitis [4]. Odontogenic OC (OOC) is a less frequent but important cause of orbital infection, with a poor prognosis. In a prior series, 45.8% of patients with OOC had final vision of light perception or worse [5].

The most common pathway of spread of OOC infection is through the paranasal sinuses; less common is the spread from premaxillary soft tissues to the orbit [6].

The difficulty of this case was that the patient manifested no odontogenic problems and did not use immunosuppressive therapies. The panoramic scan was in fact requested only after identification of the causal pathogen, because it is a commensal of the oral mucosa [7].

CST is a rare but potentially devastating complication of OOC and is secondary to thrombophlebitis in the ophthalmic vein. The valveless superior and inferior ophthalmic veins are the first portal for the extension of infection from the facial sinuses to the cavernous sinus [8].

PE could be caused by systemic infection which may induce secondary inflammatory and procoagulant responses. Inflammatory cytokines are capable of activating coagulation while thrombin can stimulate inflammatory pathways [9]. This joint cascade of inflammation and coagulation results in endovascular injury and microvascular thrombosis.

## Conclusions

The case reported illustrates the importance of a complete eye examination with careful evaluation of ocular adnexa and palpation of the periorbital area. It is also important to identify the causes of OC and systemic complications as early as possible to avoid a poor prognosis. Although our patient had only ocular signs and symptoms, the multidisciplinary approach achieved a rapid diagnosis and treatment of OOC, CST, and PE, avoiding serious and permanent sequelae such as venous brain ischemia and septicemia.

## Abbreviations

CECT: Contrast-enhanced computed tomography
CEMRI: Contrast-enhanced magnetic resonance imaging
CST: Cavernous sinus thrombosis
LE: Left eye
NCCAT: Non-contrast-enhanced computed axial tomography
OC: Orbital cellulitis
OOC: Odontogenic orbital cellulitis (OOC)
PE: Pulmonary embolism
